# Does life expectancy vary by disability status in LMICs?: A systematic review and meta-analysis

**DOI:** 10.4102/ajod.v14i0.1514

**Published:** 2025-03-12

**Authors:** Desta Debalkie Atnafu, Femke Bannink Mbazzi, Mezgebu Yitayal, Hannah Kuper

**Affiliations:** 1 International Centre for Evidence in Disability, Department of Population Health, Faculty of Epidemiology and Population Health, London School of Hygiene and Tropical Medicine, London, United Kingdom; 2 Department of Health System Management and Health Economics, School of Public Health, Bahir Dar University, Bahir Dar, Ethiopia; 3 Medical Research Council (MRC), Uganda Virus Research Institute (UVRI) and London School of Hygiene and Tropical Medicine (LSHTM) Uganda Research Unit, Entebbe, Uganda

**Keywords:** disability, life expectancy, years of life lost, LMICs, meta-analysis

## Abstract

**Background:**

People with disabilities on average experience health care barriers, poorer health and higher mortality.

**Objectives:**

This study aims to review and synthesise life expectancy (LE) and years of life lost (YLL) comparing people with disabilities to those without in low and middle-income countries (LMICs).

**Method:**

A systematic review was conducted across six databases. Longitudinal studies with a comparator group that measured LE in or YLL between people with and without disabilities in LMICs were eligible for inclusion. Two reviewers independently assessed study eligibility, extracted data and assessed the risk of bias. Meta-analyses were undertaken using R 4.3.3. The study assessed heterogeneity with I^2^ and publication bias with a funnel plot. Sub-group and meta-regression analyses were performed, and the risk of bias was evaluated.

**Results:**

Twelve full-text articles were included in this meta-analysis. The pooled mean LE was lower in people with disabilities (57.98 years; 95% confidence intervals [CI]: 53.40–62.95) compared with people without disabilities (70.86 years; 95% CI: 64.06–78.38). The overall weighted years of YLL in people with disabilities was 15.84 years (95% CI: 11.1–22.61). There was no significant difference in YLL between men (16.33 years; 95% CI: 11.49–23.21) and women (13.7 years; 95% CI: 8.45–22.22).

**Conclusion:**

The average LE in people with disabilities was substantially lower compared to those without disabilities in LMICs. This inequity highlights that health systems and public health efforts are failing to meet the needs of people with disabilities and must be improved to become more inclusive.

**Contribution:**

The study emphasises the need for inclusive policies and robust research in the health system to address health disparities.

## Introduction

Globally, there are over 1.3 billion (16%) people with disabilities (World Health Organization [WHO] 2022), with 80% residing in low- and middle-income countries (LMICs) (WHO 2022). This number is anticipated to increase with further population growth and ageing (WHO 2011). People with disabilities face a range of exclusions and adverse conditions. With respect to health, on average, they experience poorer health and higher mortality on account of a number of different pathways (Kuper & Phyllis Heydt 2019; WHO 2011, 2015). Firstly, they are on average poorer and often reside in economically impoverished settings (WHO 2011, 2022), contributing to poorer health and functioning (Centers for Disease Control and Prevention 2008). Secondly, they have a higher prevalence of comorbidities and secondary conditions resulting from their impairment, such as diabetes, stroke and pressure sores (Kuper & Phyllis Heydt 2019; WHO 2022). Thirdly, people with disabilities are more susceptible to behavioural health risks (WHO 2011), such as physical inactivity (Hollis et al. 2020), smoking (Armour et al. 2007) and obesity (Maïano et al. 2016). Fourthly, people with disabilities commonly face barriers when seeking care, including unfavourable attitudes from health care professionals (Adugna et al. 2020), high cost of medical care (Dagnachew, Meshesha & Mekonen 2021) and inaccessible facilities (Pinto et al. 2021). Consequently, they often lack adequate access to both general and disability-specific health care and rehabilitation services (Kuper & Phyllis Heydt 2019; WHO 2022), despite their greater health care needs (Kuper & Phyllis Heydt 2019). As a result of these factors, people with disabilities face an elevated risk of morbidity and mortality compared to their non-disabled peers (Lauer & McCallion 2015; Park et al. 2017).

There is growing evidence of a shorter life expectancy (LE) for people with disabilities, including in LMICs, which may be around 10–20 years (Da Roza et al. 2023; Egüez-Guevara & Andrade 2015; Keeler et al. 2010; Ma et al. 2019; Rotenberg, Smythe & Kuper 2023; Ruffieux et al. 2023; Zhan et al. 2023). This gap appears to vary based on impairment type, for instance, being particularly high in people with mental illness (28.4 years) (Fekadu et al. 2015), functional impairments (16–20 years) (Bahk, Kang & Khang 2019) and physical impairments (12.7–17.1 years). There is also variation across LMICs (Da Roza et al. 2023; Ma et al. 2019; Moreno et al. 2019; Scalfari et al. 2013), for instance, a review showed that LE gaps for bipolar disorder were greater in Africa (29 years) than in Asia (12 years) (Chan et al. 2022), and overall LMICs exhibit higher LE gaps by disability status (26.1 years) compared to upper-middle-income countries (14.6 years) (Rotenberg et al. 2023). However, the LE gap for people with disabilities has not yet been systematically reviewed in LMICs, although LE serves as a key indicator of health status, outcomes and quality of life (Britain 2001), and a proxy for health equity (Rotenberg et al. 2023). Assessing LE disparities between disabled and non-disabled individuals is important to raise public awareness of health inequities and help policymakers craft effective strategies to address health care needs and prevent avoidable mortality (Chan et al. 2023; Issifou & Pewitt 2022). Consequently, this systematic review and meta-analysis were undertaken to compare LE among people with and without disabilities in LMICs and estimate average years of life lost (YLL).

## Methods

### Protocol and registration

We searched several databases (e.g., Cochrane Library, Joanna Briggs Institute [JBI] Library and DARE database) to prevent duplications for systematic reviews and meta-analyses on the subject being studied. The study protocol was registered with PROSPERO – Prospective Register of Systematic Reviews and Meta-analysis (CRD42024499640) – and followed the Preferred Reporting Items for Systematic Reviews and Meta-Analyses (PRISMA) reporting guidelines (Online Appendix, Table 1-A1).

### Searching strategies

We used the Population, Intervention, Comparator, Outcome, Timing, and Study design (PICOT/S) framework to clarify the research parameters (Table 1).

**TABLE 1:** PICOT/S framework for systematic review and meta-analysis on the link between life expectancy and disability in low and middle-income countries.

Parameters	Characteristics
P-Population	People with and without disabilities in LMICs
I-Intervention	Not applicable
C-Comparison	People without disabilities or the general population in LMICs
O-Outcome	Life expectancy (LE) or Years of life lost (YLL)
T-Time	Studies published from January 2005 to 03 March 2023
S-Study design	All quantitative study designs

LMICs, low and middle-income countries.

We systematically searched six electronic databases: Medline, Embase, Global Health, Scopus, Web of Science and Cochrane Library for studies on 03 March 2023. Google and Google Scholar search engines assessed for grey literature and additional sources. In addition, the reference tracing of included studies was conducted and more eligible articles were obtained. The initial search was carried out in the Medline database using the search strategy string (Online Appendix, Table 2-A1). The search was conducted using keywords, vocabulary words and MeSH (medical subject headings) terms related to disability, LE and LMICs (classified by the World Bank Group) (Hamadeh et al. 2022). Boolean operators (‘OR’, ‘AND’ and ‘NOT’) as well as truncations (*) were applied both individually and collectively.

### Eligibility criteria for study inclusion

We included published articles between 01 January 2005 and 03 October 2023, benchmarking the World Health Assembly Resolution on Universal Health Coverage in 2005 and the growing momentum advocating for the adoption of the United Nations Convention on the Rights of Persons with Disabilities (UNCRPD) in 2006 (Guide 2014; Latko et al. 2011). Eligible studies had to fulfil the following criteria: (1) quantitative observational (cross-sectional, case-control, cohort) or interventional (trial) studies; (2) report and/or compare data on the mean or standard error of LE (at birth or later) between people with and without disabilities of all ages; (3) undertaken in LMICs (Hamadeh et al. 2022); (4) published in English; and (5) disability assessed using the Washington Group module and/or other reliable and/or validated measures of disability (Hanass-Hancock et al. 2023). The review excluded records with no full text, editorials, review studies or qualitative research. Studies without a clear measure of LE were excluded.

### Study outcome and explanatory variables

The primary outcome of this review was the LE of people with and without disabilities, measured as the average age at death, or YLL, measured as the mean difference in LE between people with disabilities and those without disabilities or the general population. Other measures of LE, such as subjective LE or disability-free/healthy LE, were not eligible. The LE and YLL were recorded by sex (male and female), where available.

### Study screening and data extraction strategy

After retrieving all records from the databases, we exported records to the bibliographic software, Endnote Version 20 reference manager, to remove the duplicate studies. Then, the remaining studies were double-screened (DDA and HK) using the Rayyan app based on title and abstract against criteria to identify possibly eligible studies. Full-text studies were evaluated to decide the inclusion of articles in the analysis. The disagreements in study screening were resolved through a consensus-based discussion.

The Joanna Briggs Institute (JBI) data extraction tool was applied to systematically extract and organise data, ensuring consistency and accuracy. All required data were independently extracted by two authors (DDA and HK) and recorded in a Microsoft Excel spreadsheet. The data extraction protocol includes the first author’s name, publication year, study settings/country, study design, sample size, methods of analysis used, sex of respondents, age at which LE was estimated and a measure of LE (e.g. mean LE, YLL, a measure of effect where available (e.g., 95% confidence interval [CI], *p*-value), methods of calculating LE and disability type.

### Methods of assessing the outcomes

This systematic review and meta-analysis reported LE of people with disabilities and people without disabilities or the general population. If multiple reports of LE at different set ages were available, the mean LE for the longest duration of follow-up and at the youngest set age were selected. For studies with multiple LE or YLL estimates for different impairment types, we used one estimate per study after calculating the weighted average based on the number of participants for each impairment type. For studies that provided CIs instead of standard deviations, we converted the CIs to standard deviations for the meta-analysis. If studies did not report standard deviations for LE/YLL estimates, we employed multiple imputation methods using pooled effect sizes from other studies included in the meta-analysis (Furukawa et al. 2006).

### Quality assessment of included studies

The quality of the included studies was evaluated using the JBI critical appraisal checklist (Online Appendix, Table 3-A1) (Barker et al. 2024). The JBI checklist contains 11 parameters that were listed from ‘participant ascertainment’ to ‘the appropriateness of the statistical analysis’. Two reviewers independently (DDA and HK) assessed the quality of the included studies. Disagreements among reviewers were resolved through discussion. Finally, studies with an overall quality appraisal score of ≥ 5 were included in the review.

### Data analysis and presentation

We extracted the data and exported it to R 4.3.3 statistical software (The R Project for Statistical Computing, Auckland, North Island, New Zealand) for further analysis. A random-effects model (Restricted Maximum Likelihood Methods) was employed in this meta-analysis to obtain pooled effect estimates, summarised as average LE for people with and without disabilities or the general population, and the YLL, with 95% CIs (Borenstein et al. 2010). The heterogeneity between the included articles was computed and checked using Cochrane *Q* test statistic (chi-square), *I*^2^ index and *p*-values. The heterogeneity was classified as low (25%), moderate (50%) or high (75%) based on the results of the *I*^2^ test (Higgins et al. 2003). Meta-regression and sub-group analyses (considering sex, age group and disability type) were performed respectively, using a random-effect model to investigate the sources of heterogeneity. A sensitivity analysis was conducted to assess the impact of a single study on the overall estimation of meta-analysis.

Univariable and multi-variable meta-regression analyses were performed to identify how much each study characteristic contributed to the heterogeneity in estimating the pooled YLL estimates. The multivariable meta-regression analysis included all potential moderators in the final model. This analysis aimed to measure the extent to which all moderators included in the final model explained the observed true heterogeneity (*R*^2^) and the remaining unexplained or residual heterogeneity (*I*^2^) and to assess if the model adequately explained the observed variability (Qm). Forest plots were computed to visualise the presence of heterogeneity among studies. A meta-cumulative analysis was conducted to examine the pattern of effects and the significance of cumulative effects over the publication years. The publication bias was assessed objectively using Egger’s regression and Begg’s test (Begg & Mazumdar 1994; Egger et al. 1997), and subjectively by observing the funnel plot. A *p*-value of less than or equal to 0.05 was considered statistically significant.

## Review findings

### Search results

**FIGURE 1:**
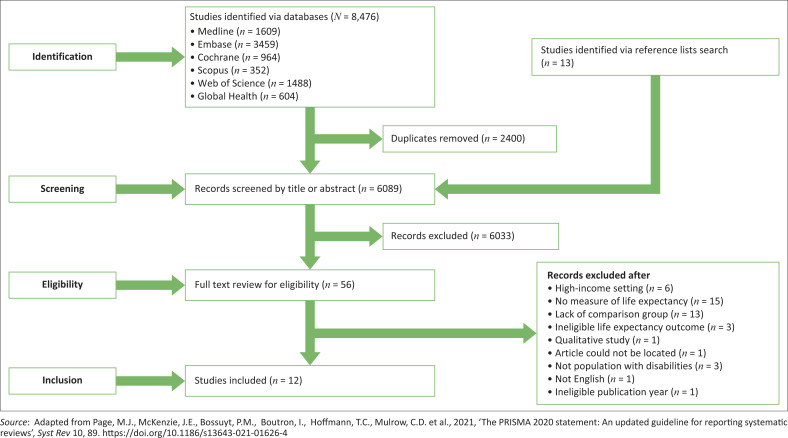
PRISMA flow chart.

Out of the initial 8476 articles retrieved on LE and disability status, 2400 records were excluded because of duplications, leaving 6089 articles for title and abstract screening. Then, 56 studies were selected for full-text screening; 44 studies were excluded (Online Appendix, Table 4-A1) leaving 12 articles that fulfilled the eligibility criteria and were included in the analysis (Figure 1).

### Characteristics of the included studies

This study extracted and analysed data from the 12 eligible studies. Sample sizes of the included studies ranged from 510 (Ran et al. 2020) to 1 359 812 (Zhan et al. 2023) participants. In terms of regional distribution, eight studies were conducted in Asia (six from China, one each from India and Mongolia), two in South America (both from Brazil) and two in Africa (Ethiopia and South Africa). All studies employed a longitudinal observational study design and were published between the years 2015 and 2023. In the included studies, participants were identified from death registers, health care records, health insurance databases, disability registers and study cohorts. Most included studies (*n* = 11) used the International Classification of Diseases (ICD) criteria for diagnosing or measuring disability. The follow-up duration varied from 2 to 24 years. In the included studies, various impairment types were evaluated, including psychosocial (*n* = 7), cognitive (*n* = 2), neurological disorders (*n* = 2) and multiple disabilities (*n* = 1) (Online Appendix, Table 5-A1). Approximately nine studies reported the average LE and the LE gaps concurrently (Table 2).

**TABLE 2:** Characteristics of included studies.

Author’s name, publication year	Country	Follow-up years	Type or measurement of disability	Number of people with disabilities	Number of people without disabilities	Age range	% Female	Number of deaths of people with disabilities	Number of deaths of people without disabilities
Andrade, F.C., 2019	Brazil	11	Cognitive (Screening Questionnaire)	147	1969	> 60	59	NA	NA
Da Roza, D.L., 2023	Brazil	15	Mental disorder (Clinical)	4019	1 328 535	> 15	45	803	NA
Fekadu, A, 2015	Ethiopia	10	Severe mental illness (Clinical)	919	67 459	15–49	38	121	NA
Liu, X., 2022	China	3.6	Schizophrenia (Clinical)	228 572	NA	> 15	52	7907	NA
Ma, Y., 2019	Mongolia	7	Neurological (Clinical)	1137	NA	> 20	37	1137	NA
Ran, M.S., 2020	China	21	Schizophrenia (Clinical)	510	123 572	> 15	54	196	NA
Ruffieux, Y., 2023	South Africa	3	Mental illness (Clinical)	282 926	787 257	15 – 85	52	10 964	21 195
Zhan, P., 2023	China	2	Multiples (Clinical)	1 359 812	75 428 900	> 20	44	49 973	NA
Ren, J., 2023	China	5	Schizophrenia (Clinical)	80 540	NA	> 15	49	9483	NA
Banerjee, T.K., 2017	India	5	Dementia (Clinical)	103	100 699	> 50	47	17	NA
Wu, J., 2023	China	11	Schizophrenia (Clinical)	18 178	NA	> 0	38	18 178	NA
Luo, Z., 2021	China	24	Neurological (Clinical)	2411	4522	> 0	46	4432	NA

NA, not available.

In terms of LE estimation methods, five studies used life table methods, three employed Chiang’s method and the remainder used other approaches (Table 3). The ages at which LE was derived varied: about six studies used age at birth, while the other studies used different set ages (e.g. 20, 60 years). The mean LE differed between people with disabilities and the reference population (e.g. total population, individuals without disabilities), ranging from 47.27 to 89.34 years and from 55.6 to 80.2 years, respectively. The YLL (or LE gaps) also varied between 2.19 and 28.4 years.

**TABLE 3:** Key results.

Author’s name, publication year	Life expectancy set age (Year)	Method of life expectancy ascertainment	Life expectancy in people with disabilities (mean, 95% CI)	Life expectancy in people without disabilities (mean, 95% CI)	Years of life lost	Risk of bias
Andrade, F.C., 2019	60	Markov chain	Overall: 62.6 Men: 61.9 Women: 63.1	Overall: 77.3 Men: 75.3 Women: 78.7	Overall: 15.4	9
Da Roza, D.L., 2023	Birth	Chiang’s method	Overall: 47.3 Men: 43.4 Women: 51.4	Overall: 75.0	Overall: 27.6 Men: 27.9 Women: 27.1	8
Fekadu, A, 2015	Birth	Chiang’s method	Overall: 49.3 Men: 47.2 Women: 56.0	Overall: 55.7 Men: 52.3 Women: 59.6	Overall: 28.4 Men: 26.3 Women: 30.7	9
Liu, X., 2022	Birth	Chiang’s method	Overall: 55.7 Men: 52.8 Women: 59.0	Overall: 76.9 Men: 74 Women: 80.3	Overall: 21.2 Men: 21.2 Women: 21.3	10
Ma, Y., 2019	Not reported	Life table	NA	NA	Overall: 19.0 Men: 18.6 Women: 18.7	7
Ran, M.S., 2020	Birth	Survival function	Overall: 55.7 Men: 50.6 Women: 58.5	Overall: 69.7 Men: 67.7 Women: 71.9	Overall: 19.0	9
Ruffieux, Y., 2023	36	Bootstrap simulation	NA	NA	Overall: 2.9 Men: 3.8 Women: 2.2	9
Zhan, P., 2023	20	Period life table	Overall: NA Men: 65.2 Women: 68.1	Overall: NA Men: 80.2 Women: 84.6	Overall: 15.7 Men: 15.0 Women: 16.5	11
Ren, J., 2023	Birth	Life table	Overall: 60.0 Men: 57.8 Women: 61.6	Overall: 74.8 Men: 72.4 Women: 77.4	Overall: 15.3 Men: 15.8 Women: 14.6	11
Banerjee, T.K., 2017	74	Life table	Overall: 89.3 Men: NA Women: NA	NA	Overall: 15.4 Men: NA Women: NA	9
Wu, J., 2023	Not reported	Trend analysis	NA	NA	Overall: 21.7 Men: NA Women: NA	9
Luo, Z., 2021	Birth	Standard reference life table	Overall: 68.4 Men: 66.3 Women: 70.9	NA	Overall: 12.1 Men: 12.4 Women: 11.8	9

NA, not available; CI, confidence interval.

### Quality appraisal

The quality scores of the included studies ranged from 7 to 11 (maximum 11 points – showing lowest possible risk of bias). Thus, all included studies were of medium to high quality, and none were rated as low (Online Appendix, Table 3-A1).

### Meta-analysis

#### The mean life expectancy in people with and without disabilities

**FIGURE 2:**
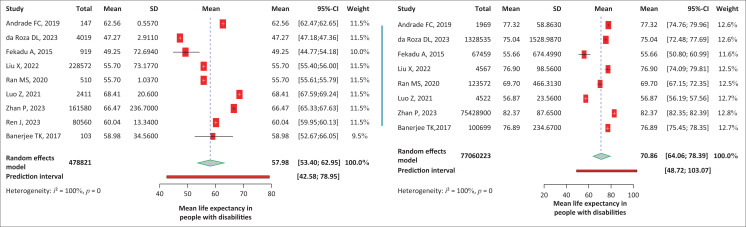
Forest plot showing the life expectancy among people with and without disabilities.

Nine studies reported LE in people with disabilities and eight in people without disabilities. The analysis estimated the average LE of people with disabilities to be 57.98 (95% CI: 53.4–62.95) years. There was significant variability in the data (*I*^2^ = 100%, *p* < 0.001), indicating a high level of heterogeneity; as a result, a random effect model was employed. For people without disabilities, the average LE was 70.86 (95% CI: 64.06–78.39), again with a high heterogeneity among studies (*I*^2^ = 100%, *p* < 0.001) (Figure 2). People with disabilities experienced a substantially lower mean LE compared to those without disabilities (-13.29 years; 95% CI: -21.58 to -5.0; *p* = 0.002) (Online Appendix, Figure 1-A1).

#### Mean years of life lost

**FIGURE 3:**
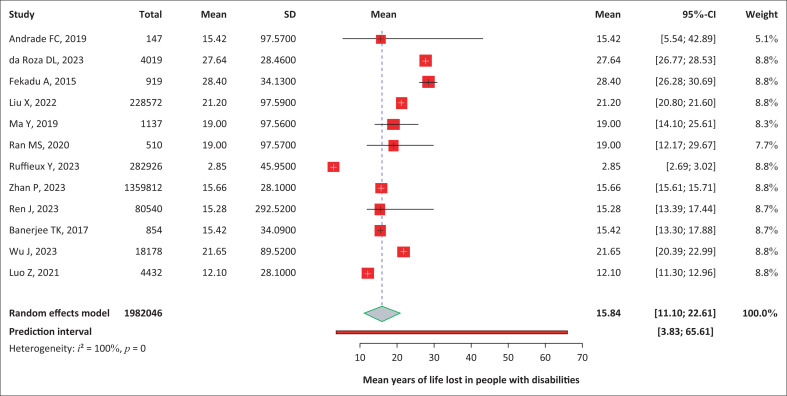
Forest plot showing the years of life lost in people with disabilities.

Eight studies presented YLL, and for the remaining four, this estimate was calculated as the mean difference in LE between people with and without disabilities. The weighted average YLL was 15.84 years (95% CI: 11.1–22.61; *I*^2^ = 99.8%, *p* < 0.001), encompassing a predictive interval from 3.83 to 65.61 years (Figure 3). It appeared that YLL was higher for men with disabilities (16.33; 95% CI: 11.49–23.21 years) compared with women with disabilities (13.70; 95% CI: 8.45–22.22) although this difference was not statistically significant (*t* = 392; *p* = 0.69) (Online Appendix, Figure 2-A1).

Publication bias and sensitivity analysis for mean years of life lost: The funnel plot displayed an asymmetrical distribution graphically, but the results of the Egger regression test (*p* = 0.79) and the Begg and Mazumdar test (*p* = 1.0) did not reach statistical significance, indicating the absence of small study effects. The trim-and-fill analysis identified and included two additional studies to address any potential oversights after examining the funnel plot and resulted in a pooled YLL estimate of 14.51 (95% CI: 10.5–20.03), with an overlap of CIs indicating a lack of statistically significant difference (Online Appendix, Figure 3-A1). A leave-one-out meta-analysis using the REML (restricted maximum likelihood) method was conducted to assess the influence of each study on the overall results. No indicated outliers confirmed robust and consistent results without single-study effects.

**FIGURE 4:**
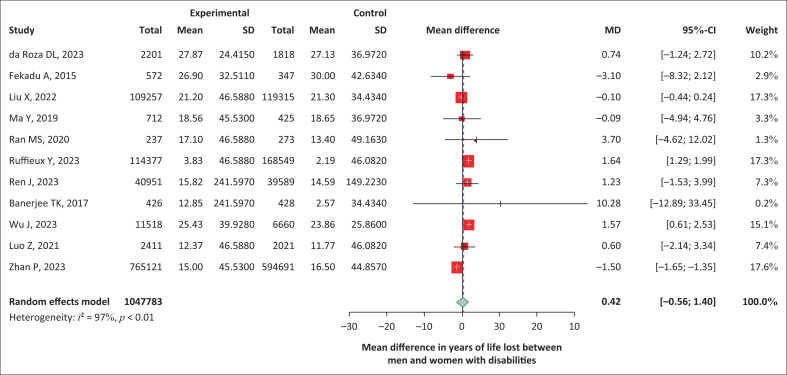
Forest plot showing the mean difference of years of life lost between men and women with disabilities.

Weighted mean differences in years of life lost by gender: There was a higher level of heterogeneity in the meta-analysis when assessing the mean difference in YLL between disabled male and female participants (*I*^2^ = 96.8%, *p* < 0.001). With a random-effects model applied, the mean difference of YLL in male versus female participants was estimated to be 0.42 (95% CI: -0.56 to 1.40) (*p* = 0.40), showing a lack of statistically significant difference in YLL by disability status between male and female participants (Figure 4).

Sub-group analysis in the weighted mean of years of life lost: The sub-group analyses were performed for different factors to identify the variation in YLL across included studies. Only the analysis stratified by WHO region revealed a statistically significant variation, as there were greater mean YLL observed in the region of the Americas at 26.06 years (95% CI:18.48–36.7) compared to the Western Pacific region at 17.2 years (95% CI:14.48–20.46) (*z* = 7.91, *p* = 0.048). Other variables, such as publication year, number of disabilities, risk of bias, study settings, disability type and follow-up duration, did not show significant heterogeneity in the pooled YLL between people with disabilities and the general population in the sub-group analysis (Online Appendix, Table 6-A1).

Meta-regression of the weighted mean of years of life lost: In the univariable meta-regression analysis, we fitted the characteristics including publication year (Qm = 0.54, *p* = 0.46), WHO region (Qm = 2.24, *p* = 0.52), length of study follow-up (Qm = 0.35, *p* = 0.55), risk of bias (Qm = 0.87, *p* = 0.35), study settings (Qm = 0.97, *p* = 0.32), disability type (Qm = 0.019, *p* = 0.99) and number of people with disabilities (Qm = 0.74, *p* = 0.39), none of which showed statistically significant association with mean YLL. Conversely, two characteristics showed significant associations with the weighted mean of YLL estimates in people with disabilities: method of LE estimation (Qm = 34.74; *p* < 0.0001) and source of data (Qm = 57.69, *p* < 0.0001) (Online Appendix, Table 7-A1). After multivariable adjustment of the above characteristics, the meta-regression, which accounted for all potential moderators, explained 76.03% (Qm = 42.06; *p* < 0.0001) of the variability in YLL (Online Appendix, Table 8-A1).

## Discussion

### Summary of key review findings

This systematic review and meta-analysis analysed 12 studies that provided quantitative data on LE and YLL among people with and without disabilities in LMICs. The findings revealed that across 9 studies, the average LE was lower in people with disabilities (57.98 years) compared to people without disabilities (70.86 years), and across all 12 studies, the YLL was approximately 16 years. There was no clear difference in LE between men and women with disabilities. Overall, there was no evidence of publication bias influencing the link between disability and YLL, and the individual studies showed a low risk of bias. The review also highlighted that no single study disproportionately influenced the collective estimation derived from the meta-analysis, suggesting a fair and equitable contribution from each study. However, sub-group analysis by data sources revealed significant differences in LE estimates, particularly larger effect size estimates from household surveys.

### Comparability with existing studies

Previous studies show that people with disabilities have a higher mortality rate and consequently are more likely to die at earlier ages compared to those without disabilities (Kuper et al. 2024; Smythe & Kuper 2024), aligning with our review. This gap arises through multiple pathways including greater poverty and marginalisation of people with disabilities and consequently poorer social determinants of health, higher risk of secondary health conditions or life-limiting conditions (Garcia-Arguello et al. 2017) and treatment side effects (e.g., metabolic syndrome caused by antipsychotics) (De Hert et al. 2012; Leung et al. 2012) or the presence of life-limiting conditions, stigma and lower health care utilisation and treatment adherence (Clement et al. 2015; Corrigan, Druss & Perlick 2014; Kuper & Phyllis Heydt 2019; WHO 2022). This stark LE gap highlights the critical need to develop disability-inclusive health system.

The LE gap in people with disabilities was also comparable with meta-analyses of YLL among people with psychosocial impairments (Chan et al. 2023; Hjorthøj et al. 2017) and across different countries globally (Laursen et al. 2013; Pan et al. 2020; Ren et al. 2023). Our findings also converge with global evidence, indicating a 10–20 years LE gap for people with disabilities, irrespective of location or type of impairment (Kuper et al. 2024). Yet, YLL in this study was higher than those meta-analyses conducted globally on bipolar disorder (Chan et al. 2022; Jayatilleke et al. 2017) and lower than findings from other studies (Laursen 2011; Laursen et al. 2013, 2016; Ren et al. 2023; Rotenberg et al. 2023; Weye et al. 2020). The differences between these studies may be because of variations in their study populations (e.g., disability type), regional differences (e.g., disparities in healthcare access, policy, socio-economic factors and lifestyle), temporal changes (e.g., improved health access over time) or differences in definition/inclusion of disability in the studies (Laursen 2011; Laursen et al. 2013).

There was no difference in YLL between men and women with disabilities in this study, in contrast to the findings of previous meta-analyses (Chan et al. 2022, 2023; Hjorthøj et al. 2017) and other primary studies (Erlangsen et al. 2017; Jayatilleke et al. 2017; Kessing, Vradi & Andersen 2015; Laursen 2011; Moreno-Küstner et al. 2021; Ren et al. 2023). This difference could be attributed to multiple factors. Firstly, equitable health care access and improved social support systems may have reduced gender disparities seen in other contexts, where women often face greater barriers (Chan et al. 2023; WHO 2011). Secondly, differences in disability type and comorbidity profiles in our reviews may have minimised variability between genders, as some conditions affect men and women similarly (Thakral, Lacroix & Molton 2019). Thirdly, prior studies highlighting gender differences often emphasised regions or populations with distinct risk factors, such as higher male mortality from risk-taking behaviours or poorer health-seeking behaviours (Erlangsen et al. 2017; Jayatilleke et al. 2017). Fourthly, methodological differences, such as smaller sample sizes in earlier studies, may have exaggerated disparities (Hjorthøj et al. 2017; Laursen 2011). Finally, historical contexts reflected in meta-analyses and older studies may no longer align with current trends because of evolving social and health care landscapes (Moreno-Küstner et al. 2021; Shi 2015).

### Strengths and limitations of the study

This is the first systematic review of the link between disability and LE in LMICs. Our approach was characterised by rigorous methodologies, including the pre-registration of protocols, adherence to PRISMA guidelines for systematic reviews, extensive searches conducted across multiple databases and the implementation of dual assessments at every stage of the review process. While our search strategy did not include studies from grey literature, there was little evidence that publication bias influenced the summary of this meta-analysis. The overall quality of the included studies was high, and the risk of bias did not significantly affect the association between disability and LE/YLL.

However, this meta-analysis is not without limitations. Our literature search for English language publications may have caused us to miss important studies. All the studies, except for one, focussed on people with psychiatric or neurological conditions which could limit the generalisability of the findings (e.g., psychotropic medication may have impacts on LE). Over half of the studies were from China, and data were missing from two WHO regions, suggesting that the studies in our review might not fully represent all LMICs, potentially limiting the generalisability of our findings. We could not carry out stratified analyses by disability type and across countries because of few eligible studies, overlooking significant variations between different subpopulations of people with disabilities. Moreover, all but one of the included studies used biomedical measures of disability based on ICD criteria despite the importance of measuring functioning including physical, sensory and psychosocial functioning. We did not consider the severity of disability and so could not assess the presence of a dose-response relationship with LE. Most of the included studies relied on health system records not initially intended for research and may have missed important factors. Furthermore, a few studies faced challenges in finding an appropriate comparison group, whether among individuals with disabilities or the general population. There was high heterogeneity in the meta-analysis for LE and YLL, likely because of limited reporting of characteristics, as meta-regression could not fully account for variability, suggesting other unknown factors.

### Implications for research, policy and practice

Our study highlights that individuals with disabilities experience a marked reduction in LE. This underscores the pressing need for policy reforms aimed at providing inclusive health care services and addressing social determinants of health, particularly in LMICs. Therefore, it is imperative for the global disability community to advocate for affordable and inclusive health care systems that can improve the well-being, quality of life and societal integration of people with disabilities.

There are also implications for research. There is a need for more consistent promotion of the bio-psycho-social model of disability view with assessment of disability through functional differences, and production of disaggregated data by disability type, as underlined in the UNCRPD declaration. Overall, we only identified 12 studies on this important topic, showing that more evidence is needed, including on the gap between male and female individuals with disabilities.

## Conclusion

Disability substantially reduced LE in LMICs. This inequity in YLL shows that health systems are failing to provide inclusive services, and therefore, interventions are needed to promote disability-inclusive health care aligned with global health targets. Moreover, this disparity in the shorter LE of people with disabilities highlighted the need for targeted policy interventions and focussed research efforts across disability types, sex and global regions. By addressing these disparities through inclusive policies and robust research, it is possible to improve health outcomes and extend the LE of people with disabilities, thereby promoting equity and social justice.
